# Supplementary material for: Epidermal Collagen Reduction Drives Selective Aspects of Aging in Sensory Neurons
Source: Aging Cell. 2024 Dec 27;24(4):e14459. doi: 10.1111/acel.14459 (PMC11984697; doi:10.1111/acel.14459)
Supplement: Supplementary file 1 — Data S1. [file ACEL-24-e14459-s001.pdf]

Supplemental Materials

Epidermal Collagen Reduction Drives Selective Aspects of Aging in Sensory Neurons

Meera M. Krishna[1,2], Swapnil G. Waghmare[1,2], Ariel L. Franitza[2], Emily C. Maccoux[2], Lezi E[1,2,*]

[1]Department of Cell Biology, Neurobiology and Anatomy, Medical College of Wisconsin, 8701 W Watertown Plank Road, Milwaukee, WI 53226, United States of America

[2]Neuroscience Research Center, Medical College of Wisconsin, 8701 W Watertown Plank Road, Milwaukee, WI 53226, United States of America

[*]Corresponding author/Lead contact:
lezie@mcw.edu
+1-414-955-2248

Running Title: Epidermal Collagen Reduction Drives Neuronal Aging

SUPPLEMENTAL FIGURE LEGENDS

Figure S1: Characterization of the aging PVD neuron

(a) Schematic diagram of the PVD neuron in control (CT) animals. The black box indicates the zone presented in the representative image of CT animals at Day 11 (D11) of adulthood. Yellow arrowheads indicate neuritic beads. Scale bar = 50 μm.

(b) Correlation between PVD beading severity and harsh touch response in CT [*mec-4(u253)*; *F49H12.4::GFP(wdIs51)*] animals at D7. See Methods for scoring criteria. *n* = 18.

(c-f) Number of dorsal *(c)* and ventral *(d)* 5° dendrites, and dorsal *(e)* and ventral *(f)* 6° dendrites in CT [*F49H12.4::GFP(wdIs51)*] animals at D3 (Dorsal: *n* = 50, Ventral: *n* = 55), D6 (Dorsal: *n* = 44, Ventral: *n* = 54), and D9 (Dorsal: *n* = 47, Ventral: *n* = 53).

(g) 6° dendrites in CT and *daf-16(mu86)* mutant animals at D3 and D7 (CT D3: *n* = 51, CT D7: *n* = 70, *daf-16* mutant D3: *n* = 47, *daf-16* mutant D7: *n* = 46). Experiments performed without FUDR.

(h-i) 5° *(h)* and 6° *(i)* dendrites in CT (*n* = 13) and *mev-1(kn1)* (*n* = 10). Experiments performed without FUDR.

(j) 5° dendrites in CT (*n* = 49) and *daf-2(e1370)* (*n* = 51).

(k-l) 5° *(k)* and 6° *(l)* dendrites normalized to body length in CT (*n* = 11) and *dpy-5(e907)* (*n* = 10).

(m-n) 5° *(m)* and 6° *(n)* dendrites normalized to body length in CT (D3: *n* = 28, D9: *n* = 51) and *col-120(sy1526)* (*n* = 25).

ns—not significant, * *p*<0.05, ** *p*<0.005, *** *p*<0.001, **** *p*<0.0001.

Figure S2: Cytoskeletal composition in PVD dendrites

(a) Split channel and merged images of microtubule localization in the PVD neuron at D7 in control (CT) animals [*ser-2(3)p::EMTB::GFP* + *ser-2(3)p::mCherry-PH(lxyEx48)*]. Red channel displays the PVD neuron, while the green channel displays the microtubules in the PVD.

(b) Split channel representative time-lapse images of actin dynamics in higher order dendrites during an 8-minute session at 2-minute intervals at D1 and D9 of adulthood in CT animals [*ser-2(3)p::GFP::UtrCH* + *ser-2(3)p::mCherry-PH(lxyEx51)*]. Red channel displays the PVD neuron, while the green channel displays the actin in the PVD.

Figure S3: Aging-associated dendritic branching correlated with proprioceptive deficits and neuritic beading

(a) Correlation between number of higher-order dendrites (5° and 6°) and harsh touch response in *mec-4(u253)* mutant animals at D7. Harsh touch response was scored based on the distance moved with a cumulative score ranging from 0 to 10, indicating impaired to normal response respectively. *n* = 18.

(b-c) Correlation between higher-order branching and the extent of neuritic beading in CT animals at D8 (*n* = 11) and D9 (*n* = 11). Higher-order dendrites are 5° dendrites *(b)* and 6° dendrites *(c)*. Beading severity is measured as 0—normal ,1—mild, 2—moderate, and 3—severe.

Figure S4: Characterization of cuticular collagens in aging

(a) (Left) Volcano plot depicting changes in expression of skin collagens in D10 animals relative to L4 animals from the GSE176088 dataset. *col-120, col-129, col-141, dpy-5, dpy-10, and rol-6* are shown in orange. (Right) qPCR verification of *dpy-5* and *col-120* mRNA expression levels in CT animals at D1 and D7, represented as relative quantity, with *ama-1* or *cdc-42* as housekeeping genes.

(b-c) Quantification of 5º *(b)* and 6º *(c)* dendrites in empty vector (EV) (*n* = 16) and *rol-6* RNAi (*n* = 6).

(d-e) 5º *(d)* and 6º *(e)* dendrites in EV (*n* = 19) and *col-129* RNAi (*n* = 20).

(f-g) 5º *(f)* and 6º *(g)* dendrites in CT and *col-141(gk5185)* mutant (*n* = 10).

(h-i) 5º *(h)* and 6º *(i)* dendrites in CT and *dpy-10(e128)* mutant (*n* = 50).

(j) mRNA levels of *dpy-5* and *col-10* (predicted paralog of *dpy-5*) after *dpy-5* RNAi clone 1 treatment *(left),* and mRNA levels of *col-120* and *dpy-3* (predicted paralog of *col-120*) after *col-120* RNAi clone 1 treatment *(right)*, relative to empty vector (EV), with *ama-1* or *cdc-42* as housekeeping genes.

(k) Comparison of body lengths between control (CT, *n* = 13) and *dpy-5(e907)* (*n* = 9).

(l) Comparison of body lengths between control (CT, *n* = 28) and *col-120(sy1526)* (*n* = 25).

(m) Summary data of lifespan analysis of CT, *dpy-5(e907)* mutant and *col-120(sy1526)* mutant animals.

ns—not significant, * $p<0.05$, ** $p<0.005$, *** $p<0.001$, **** $p<0.0001$.

Figure S5: Characterization of loss of *dpy-5* and *col-120*

(a) Quantification of 6° dendrites in CT(n = 50), *dpy-5(e907)* mutant (n = 50), and endogenous promoter-driven rescue (n = 49).

(b) 6° dendrites in CT, *dpy-5* mutant, and epidermis-specific rescue driven by *dpy-7p* (n = 60; line 1).

(c) 6° dendrites in CT (n = 40), *col-120(sy1526)* (n = 38), and endogenous promoter-driven rescue (n = 38).

(d) 6° dendrites in CT (n = 82), *col-120* mutant (n = 100), and epidermis-specific rescue driven by driven by *dpy-7p* and *col-19p* (n = 55; line 1).

(e-f) 5° *(e)* and 6° *(f)* dendrites in CT, *dpy-5* mutant, and epidermis-specific rescue driven by *dpy-7p* (n = 20; line 2).

(g-h) 5° *(g)* and 6° *(h)* dendrites in CT, *col-120* mutant, and epidermis-specific rescue driven by driven by *dpy-7p* and *col-19p* (n = 81; line 2).

(i-j) 5° *(I)* and 6° *(j)* dendrites in CT, *dpy-5* mutant, and *col-120* mutant (n = 20 for all groups).

(k-l) 5° *(k)* and 6° *(l)* dendrites in empty vector (EV) treated animals (n = 30 for both groups).

(m) 6° dendrites in EV and *dpy-5* RNAi clone 1 treated animals (n = 88 for both groups).

(n) 6° dendrites in EV and *col-120* RNAi clone 1 treated animals (n = 70 for both groups).

(o) Representative images of adulthood EV-treated, adulthood *dpy-5* clone 1 RNAi-treated, lifelong *dpy-5* clone 1 RNAi-treated, and *dpy-5* mutant animals at D3. Scale bar = 500 μm.

(p-q) 5° *(p)* and 6° *(q)* dendrites in EV and *dpy-5* RNAi clone 2 animals (n = 70 for both groups).

(r-s) 5° *(r)* and 6° *(s)* dendrites in EV and *col-120* RNAi clone 2 animals (n = 30 for both groups).

(t-u) Amplitude CV *(t)* and wavelength CV *(u)* in CT D3 (n = 28), CT D9 (n = 51), and *col-120* mutant (n = 25) at D3.

ns—not significant* p<0.05, ** p<0.005, *** p<0.001, **** p<0.0001.

Figure S6: Overexpression of epidermal collagens

(a) Quantification of 6° dendrites in control (CT, *n* = 46) and *dpy-5* epidermis-specific overexpression driven by *col-19p* (*n* = 50).

(b) 6° dendrites in CT (*n* = 53) and *col-120* epidermis-specific overexpression driven by *col-19p* (*n* =54; line 2).

(c-d) 5° *(c)* and 6° *(d)* dendrites in CT (*n* = 20) and *col-120* epidermis-specific overexpression driven by *col-19p* (*n* = 18; line 1).

(e) Summary of lifespan analysis of CT, *dpy-5* epidermis-specific overexpression, and *col-120* epidermis-specific overexpression line 1 animals without FUDR. Replicates in main figure indicated in bold. Same transgenic strains as in *(a)* and *(c-d)*.

(f-i) Mean amplitude *(f)*, and wavelength *(g)* normalized to control, amplitude CV *(h)*, and wavelength CV *(i)* in CT (*n* = 23) in *dpy-5* overexpression (same transgenic strain as in *(a)*) (*n* = 21).

ns—not significant, * $p<0.05$, *** $p<0.001$, **** $p<0.0001$.

Figure S7: Interactions between *rig-3*/IgSF, collagen genes, and *daf-16*/FOXO

(a-b) Quantification of 5º *(a)* and 6º *(b)* dendrites in empty vector (EV, *n* = 208), *ina-1* RNAi (*n* = 80), *ddr-2* RNAi (*n* = 30), *sdn-1* RNAi (*n* = 30), *pat-2* RNAi treatment (*n* = 92).

(c) 6º dendrites in EV and *rig-3* RNAi treated CT animals (*F49H12.4::GFP(wdIs51)*) *(n* = 84).

(d) 6º dendrites in CT (*ser-2(3)p::GFP(lxyEx77)*) (*n* = 91) and *rig-3(ok2156)* mutant (*n* = 90).

(e-f) 5º *(e)* and 6º *(f)* dendrites in CT and *rig-3* mutant (*n* = 10).

(g) 6º dendrites in CT (*n* = 37), *rig-3* mutant (*n* = 40), and pan-neuronal rescue (*n* = 37) (driven by *unc-33p*).

(h) 6º dendrites in CT (*n* = 41), *rig-3* mutant (*n* = 46), and interneuron rescue (*n* = 37) (driven by *nmr-1p*).

(i) Expression levels of *rig-3* and *nmr-1* in neuronal tissues of wild-type animals (Wormbase).

(j) 6º dendrites in *dpy-5(e907)* mutant animals treated with EV (*n* = 84) or *rig-3* RNAi (*n* = 85), as well as in *col-120(sy1526)* mutant animals treated with EV (*n* = 88) or *rig-3* RNAi (*n* = 86).

(k) 6º dendrites in CT (*n* = 76), *rig-3* pan-neuronal overexpression driven by *unc-33p* (*n* = 57), *rig-3* overexpression in *dpy-5* mutant (*n* = 10), and *rig-3* overexpression in *col-120* mutant (*n* = 37).

(l) 6º dendrites in CT (*n* = 30), *dpy-5* epidermis-specific overexpression driven by *col-19p* (*n* = 22), and *dpy-5* overexpression in *rig-3* mutant (*n* = 18).

(m) 6º dendrites in CT (*n* = 10), *col-120* epidermis-specific overexpression driven by *col-19p* (*n* = 19; line 1), and *col-120* overexpression in *rig-3* mutant (*n* = 20).

(n) KEGG pathway analysis between control and *dpy-10* mutant animals (GSE19310).

(o-p) 5º *(o)* and 6º *(p)* dendrites in CT (*n* = 12), *col-120* epidermis-specific overexpression driven by *col-19p* (*n* = 13; line 1), and *col-120* overexpression line 1 in *daf-16(mu86)* mutant background (*n* = 13). Experiment performed without FUDR.

ns—not significant, * $p<0.05$, ** $p<0.01$, *** $p<0.001$, **** $p<0.0001$.

Table S1: Comparison of branching in PVDL and PVDR, and Dorsal, Ventral, Anterior, and Posterior sections of PVD neuron

Difference between medians for CT animals at D3 and D9 with FUDR, and D3 and D7 without FUDR. ns—not significant, * $p<0.05$, ** $p<0.01$, *** $p<0.001$, **** $p<0.0001$.

Table S2: Correlation between excessive PVD dendritic branching and proprioceptive deficits

D3 and D9 CT animals were assayed and combined for analysis when FUDR was used for age synchronization; Data from D3 and D7 CT animals were collected when assays were conducted without FUDR. ns—not significant,* $p<0.05$, ** $p<0.01$, *** $p<0.001$, **** $p<0.0001$.

Table S3: Strain information

Table S4: Plasmid information

Table S5: Primer information

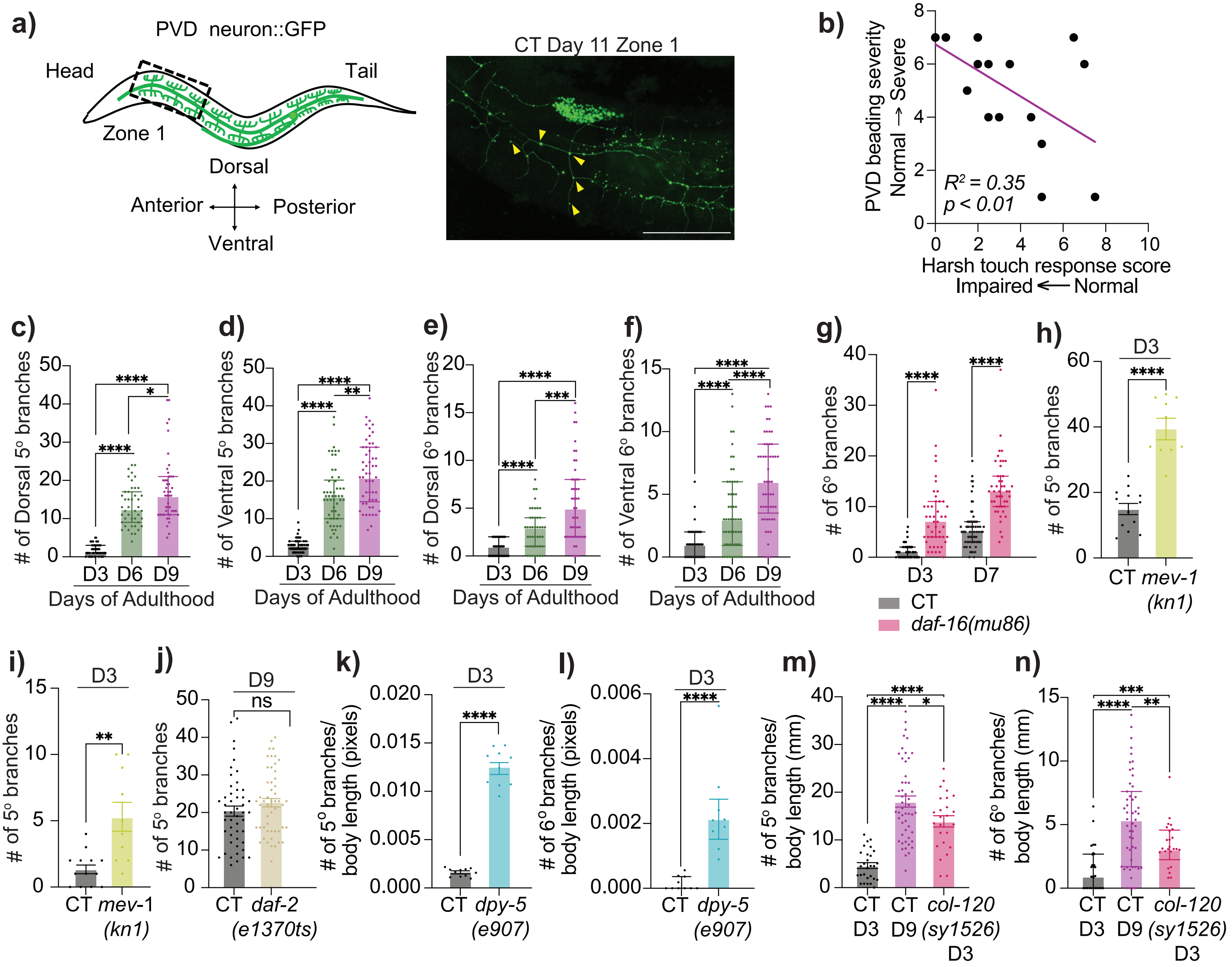
a)
PVD neuron::GFP
Head
Tail
Zone 1
Dorsal
Anterior
Posterior
Ventral
CT Day 11 Zone 1
b)
PVD beading severity
Normal → Severe
$R^2 = 0.35$
$p < 0.01$
Harsh touch response score
Impaired ← Normal
c)
# of Dorsal 5° branches
Days of Adulthood
D3 D6 D9
d)
# of Ventral 5° branches
Days of Adulthood
D3 D6 D9
e)
# of Dorsal 6° branches
Days of Adulthood
D3 D6 D9
f)
# of Ventral 6° branches
Days of Adulthood
D3 D6 D9
g)
# of 6° branches
D3 D7
CT
daf-16(mu86)
h)
D3
# of 5° branches
CT mev-1 (kn1)
i)
D3
# of 5° branches
CT mev-1 (kn1)
j)
D9
ns
# of 5° branches
CT daf-2 (e1370ts)
k)
D3
# of 5° branches/ body length (pixels)
CT dpy-5 (e907)
l)
D3
# of 6° branches/ body length (pixels)
CT dpy-5 (e907)
m)
# of 5° branches/ body length (mm)
CT D3
CT D9
col-120 (sy1526) D3
n)
# of 6° branches/ body length (mm)
CT D3
CT D9
col-120 (sy1526) D3

Figure S1

a)

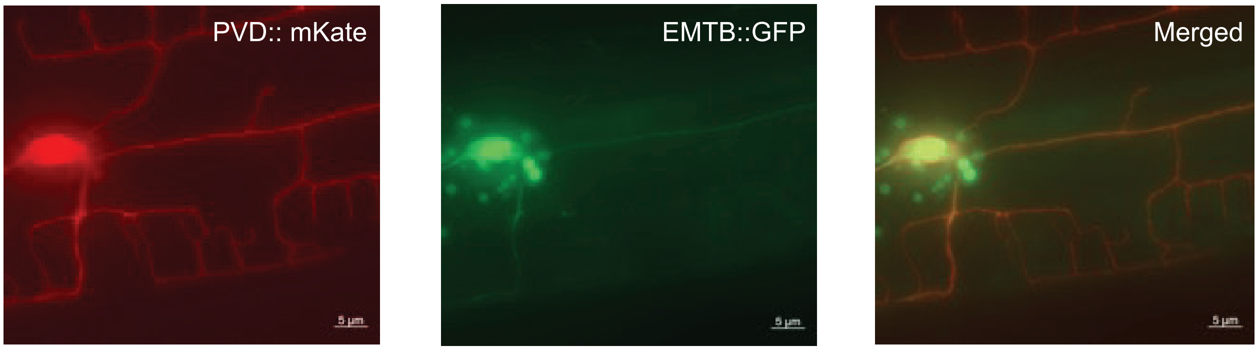
PVD:: mKate
EMTB::GFP
Merged
5 µm
5 µm
5 µm

b)

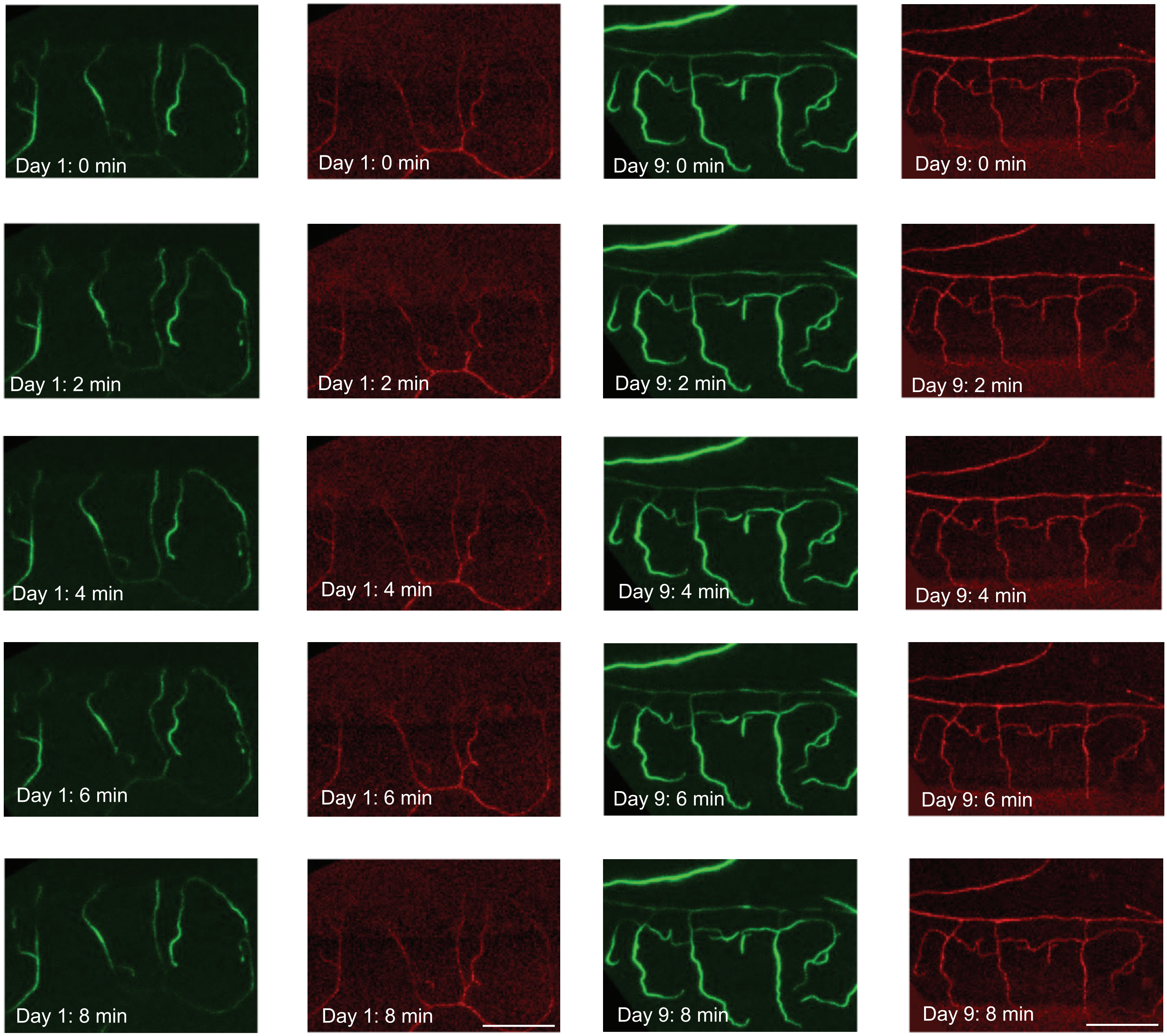
Day 1: 0 min
Day 1: 0 min
Day 9: 0 min
Day 9: 0 min
Day 1: 2 min
Day 1: 2 min
Day 9: 2 min
Day 9: 2 min
Day 1: 4 min
Day 1: 4 min
Day 9: 4 min
Day 9: 4 min
Day 1: 6 min
Day 1: 6 min
Day 9: 6 min
Day 9: 6 min
Day 1: 8 min
Day 1: 8 min
Day 9: 8 min
Day 9: 8 min

Figure S2

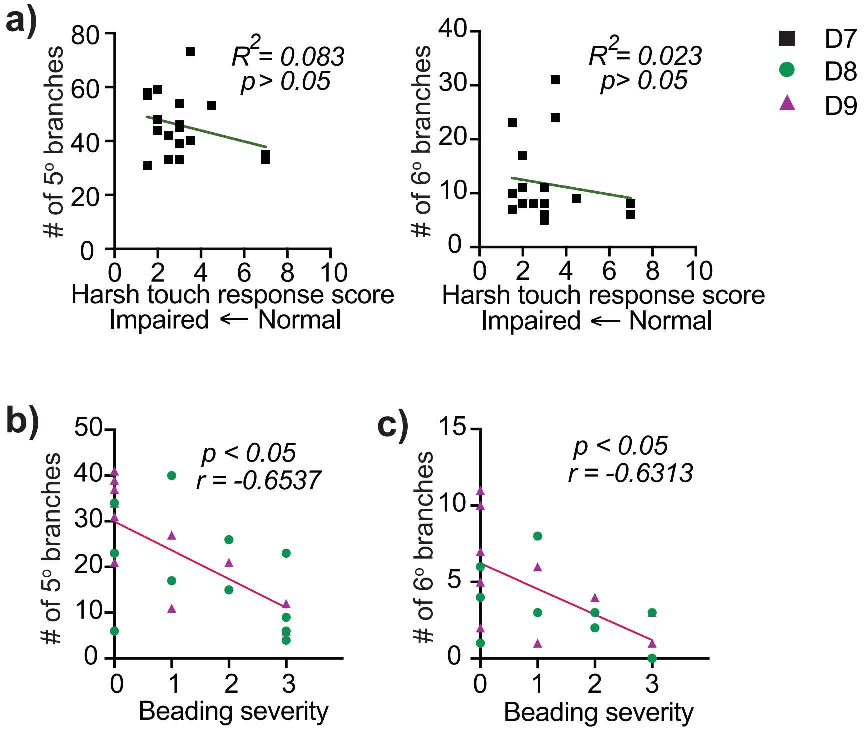
a)
80
60
40
20
0
# of 5° branches
$R^2 = 0.083$
$p > 0.05$
0 2 4 6 8 10
Harsh touch response score
Impaired ← Normal
40
30
20
10
0
# of 6° branches
$R^2 = 0.023$
$p > 0.05$
0 2 4 6 8 10
Harsh touch response score
Impaired ← Normal
D7
D8
D9
b)
50
40
30
20
10
0
# of 5° branches
$p < 0.05$
$r = -0.6537$
0 1 2 3
Beading severity
c)
15
10
5
0
# of 6° branches
$p < 0.05$
$r = -0.6313$
0 1 2 3
Beading severity

Figure S3

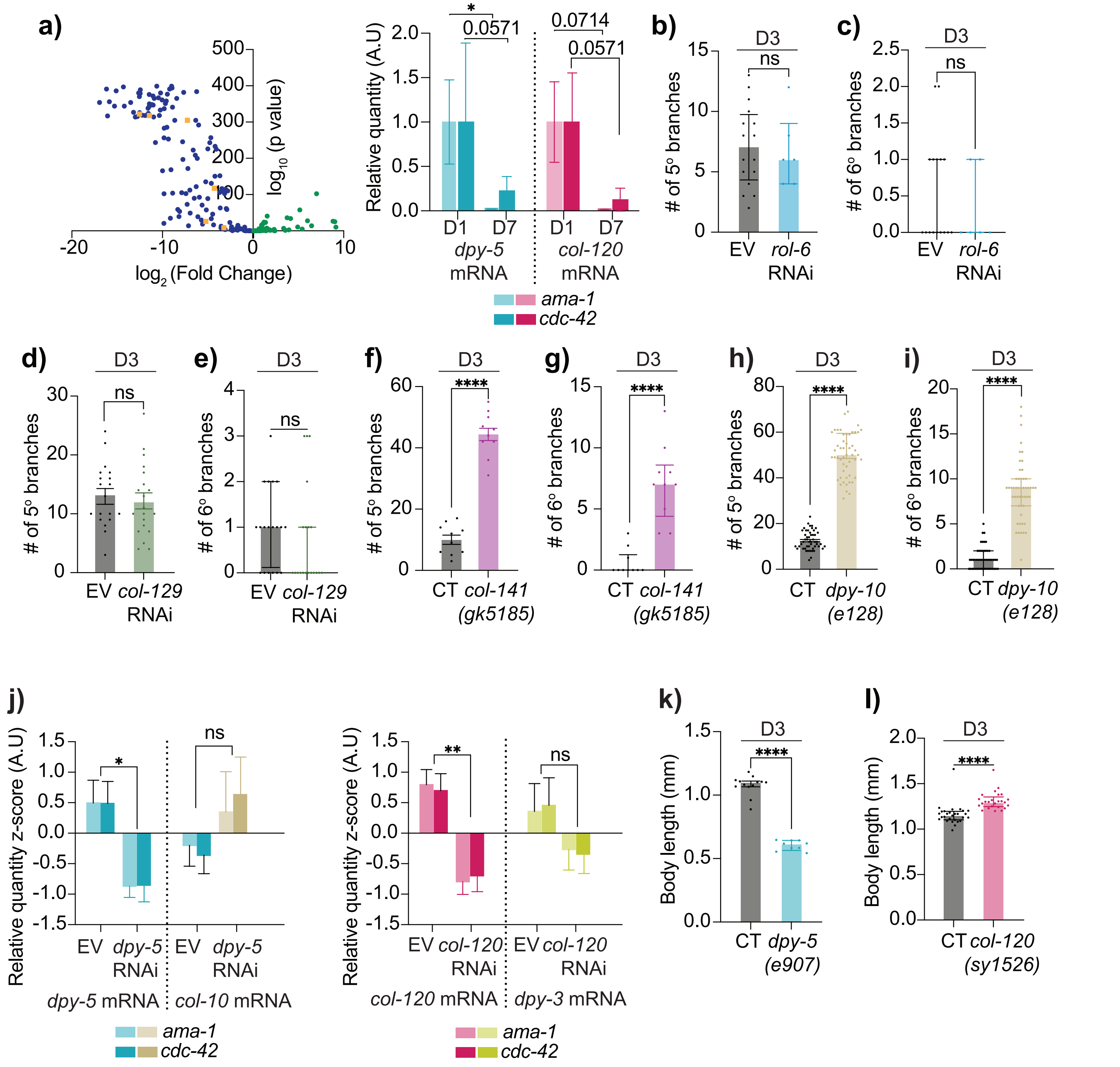

m)

Comparison	Median Lifespan	Maxiumum Lifespan	Significance vs CT		Starting *n*	Censored *n*
			dpy-5 mutant	*col-120* mutant		
**CT; *dpy-5* mutant; *col-120* mutant rep 1**	**18; 15.5; 14**	**29; 16; 18**	**0.0883**	*****	**30; 30; 30**	**7; 14; 12**
CT; *dpy-5* mutant; *col-120* mutant rep 2	22; 18; 15	25; 15; 22	*	****	30; 30; 30	4; 14; 7
CT; *dpy-5* mutant; *col-120* mutant rep 2	20; 11.5; 14	25; 16; 23	***	**	30; 30; 30	4; 15; 8

Figure S4

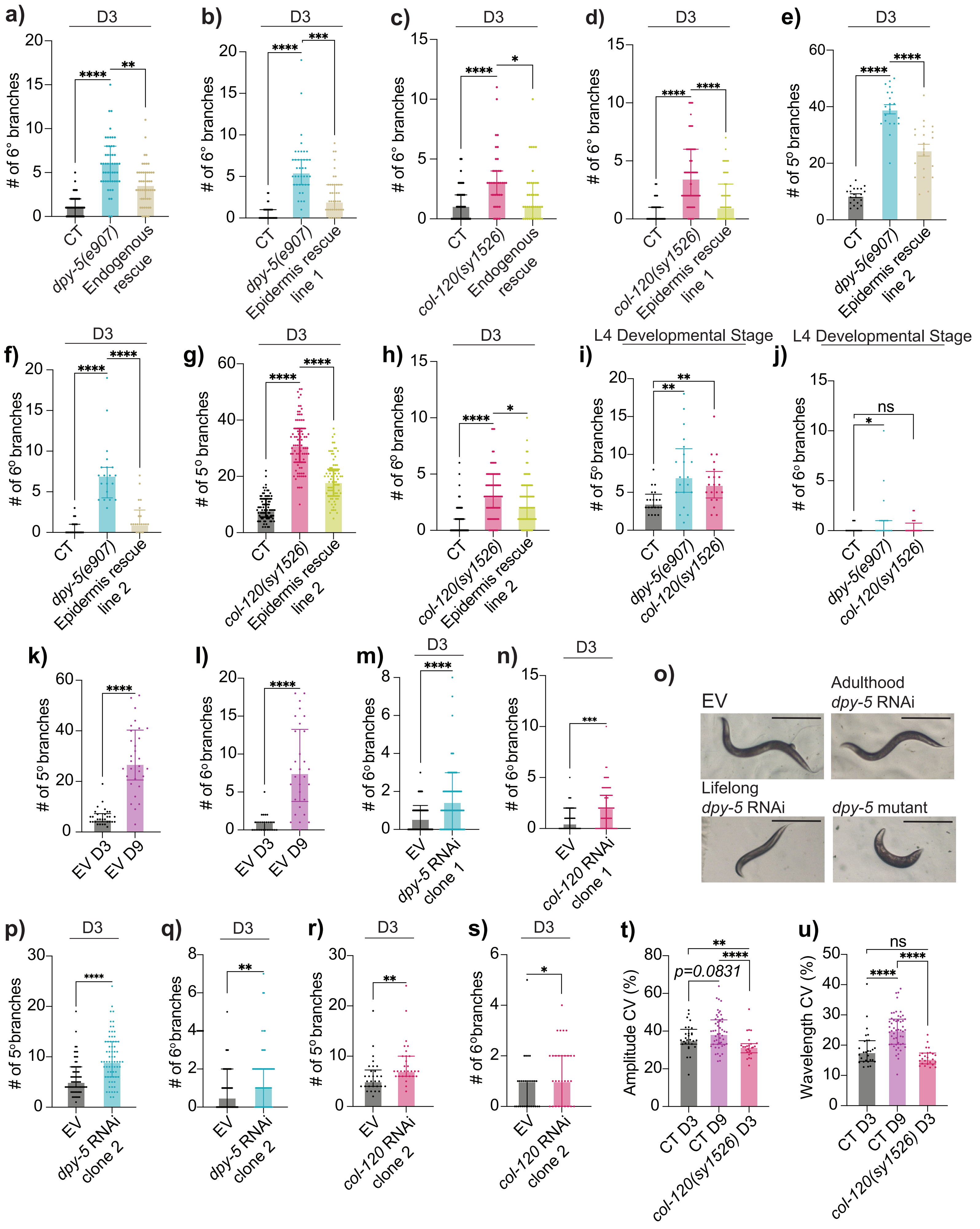
a)
D3
# of 6° branches
CT
dpy-5(e907)
Endogenous rescue
b)
D3
# of 6° branches
CT
dpy-5(e907)
Epidermis rescue line 1
c)
D3
# of 6° branches
CT
col-120(sy1526)
Endogenous rescue
d)
D3
# of 6° branches
CT
col-120(sy1526)
Epidermis rescue line 1
e)
D3
# of 5° branches
CT
dpy-5(e907)
Epidermis rescue line 2
f)
D3
# of 6° branches
CT
dpy-5(e907)
Epidermis rescue line 2
g)
D3
# of 5° branches
CT
col-120(sy1526)
Epidermis rescue line 2
h)
D3
# of 6° branches
CT
col-120(sy1526)
Epidermis rescue line 2
i)
L4 Developmental Stage
# of 5° branches
CT
dpy-5(e907)
col-120(sy1526)
j)
L4 Developmental Stage
# of 6° branches
ns
CT
dpy-5(e907)
col-120(sy1526)
k)
# of 5° branches
EV D3
EV D9
l)
# of 6° branches
EV D3
EV D9
m)
D3
# of 6° branches
EV
dpy-5 RNAi clone 1
n)
D3
# of 6° branches
EV
col-120 RNAi clone 1
o)
EV
Adulthood dpy-5 RNAi
Lifelong dpy-5 RNAi
dpy-5 mutant
p)
D3
# of 5° branches
EV
dpy-5 RNAi clone 2
q)
D3
# of 6° branches
EV
dpy-5 RNAi clone 2
r)
D3
# of 5° branches
EV
col-120 RNAi clone 2
s)
D3
# of 6° branches
EV
col-120 RNAi clone 2
t)
Amplitude CV (%)
p=0.0831
CT D3
CT D9
col-120(sy1526) D3
u)
Wavelength CV (%)
ns
CT D3
CT D9
col-120(sy1526) D3

Figure S5

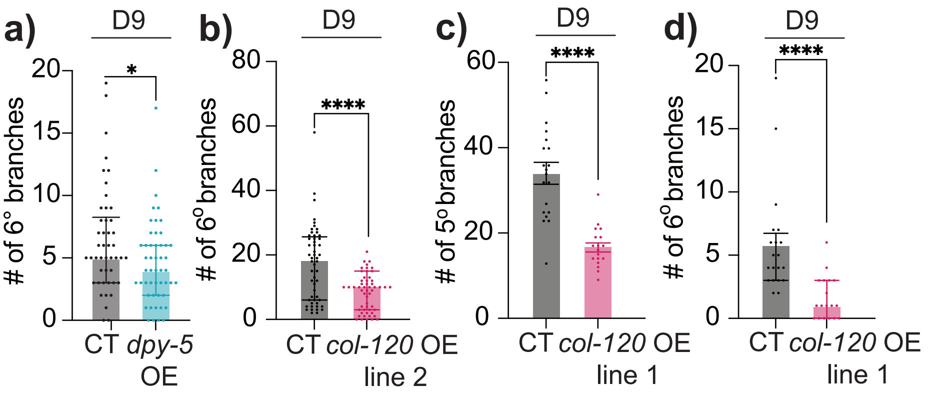

e)

Comparison	Median Lifespan	Maximum Lifespan	Significance	Starting *n*	Censored *n*
CT vs *dpy-5* OE rep 1	11; 11	20; 18	ns	63; 63	37; 25
**CT vs *dpy-5* OE rep 2**	**11; 11**	**16; 16**	**ns**	**100; 100**	**60; 38**
CT vs *dpy-5* OE rep 3	12; 10	15; 15	ns	100; 100	49; 32
CT vs *col-120* OE rep 1	11; 9	18; 16	*	72; 72	42; 39
CT vs *col-120* OE rep 2	9; 8	15; 15	ns	100; 100	62; 51
**CT vs *col-120* OE rep 3**	**8; 8**	**14; 14**	**ns**	**100; 100**	**60; 41**

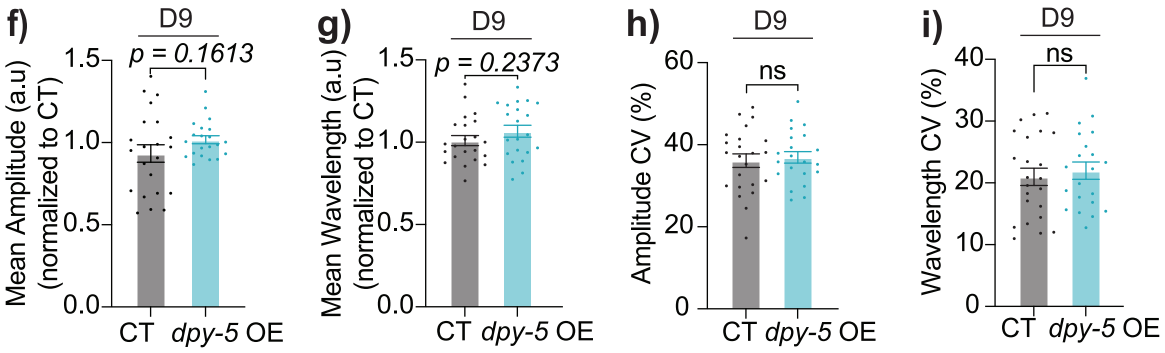

Figure S6

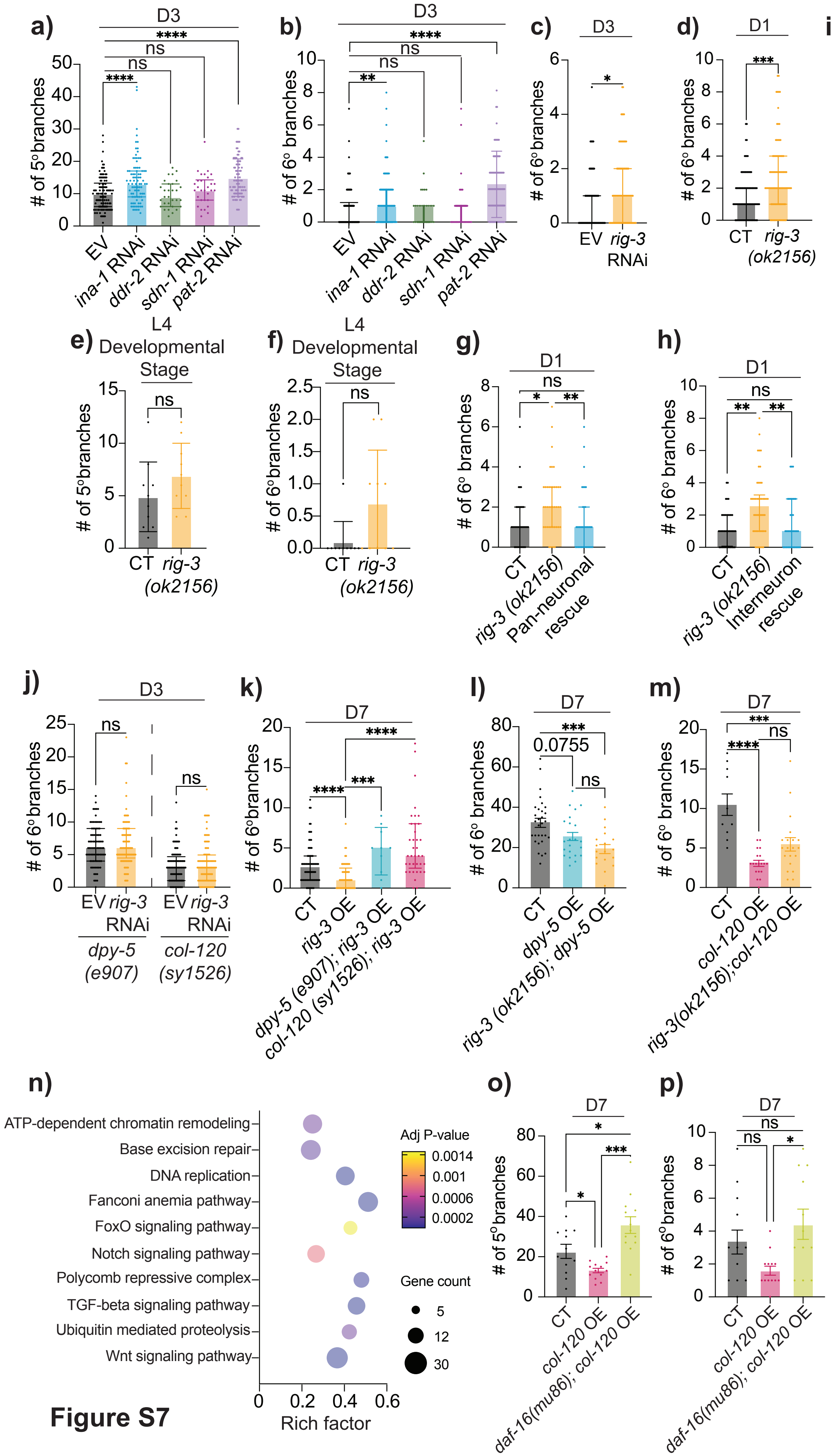

i)

rig-3 expression

Cell type	Cells (%) Expressing	Transcripts Per Million (TPM)
I4	53.03468	1052.135
I3	50	1067.467
NSM	43.00699	630.9949
PDB	40.67797	309.6697
DB	38.72153	420.6172
I1	37.37374	932.6668
AVA	35.364042	529.0081
RIH	35.2459	474.0346
MI	31.0536	396.0204
DA	29.56044	348.7844
VB01	29.26739	237.1305
AVD	28.057554	482.8923
M3	26.92308	601.7634
M4	25	335.4531
ASER	23.268698	356.8324
AVE	20.713578	332.7621
HSN	20.43011	235.2533
DB01	19.46903	144.9106
M1	18.39623	117.862
AVM	17.30769	84.1889
SMB	15.99424	112.7572
I5	14.61988	91.1973
AVB	13.744076	138.4513
AQR	13.533835	205.7389
M5	13.4715	179.0879
AVH	13.01587	111.586
RIR	12.88344	297.6041
SMD	12.72628	134.7581
RMD_DV	12.60097	203.7688
VA	11.13831	124.6461
VD_DD	10.62295	211.2284
SIB	9.816754	115.6844
PVC	9.448819	82.1599
RIA	9.393451	113.7795
AS	9.0771558	159.8174
URB	8.571429	52.8631
I6	8.064516	351.5068
AWC_ON	7.924528	80.3531
AVF	7.913669	71.6413
ASEL	7.1794872	80.7719
RID	7.148888	31.3134
VB	7.075472	70.1037
VB02	6.998088	54.4419
AIZ	6.867356	47.4251
PLN	6.688963	66.0832
DVA	6.557377	81.8664
RMD_LR	6.372549	85.9478
M2	6.113033	86.5894
AWC_OFF	5.857741	70.2421
ALA	5.462185	22.761
IL2_DV	5	39.6587
AIB	4.922516	27.7879
VA12	4.878049	83.8366
MC	4.849885	154.2685
RIB	4.48	78.3109
SAB	4.081633	91.8373
SAA	4.040404	72.0016
AVJ	3.926941	48.8293
IL2_LR	3.529412	18.8917
OLQ	3.529412	57.6456
IL1	3.448276	128.3607
ASH	2.675585	41.7272
BDU	2.650602	18.0707
AFD	2.448979	28.9723
RIP	2.380952	30.7552
RMF	2.290076	20.8604
PVT	2.272727	12.8807

nmr-1 expression

Cell type	Cells (%) Expressing	Transcripts Per Million (TPM)
PVC	23.62205	378.31377
AVD	21.582734	401.18426
AVA	15.89896	202.41909
AVE	14.370664	224.85122
AVG	12.41535	131.0069
RIM	9.52381	89.8226
AVF	3.23741	60.86642
SIB	2.356021	27.08129
RIP	1.190476	14.24181

Figure S7

Table S1

Comparison	with FUDR			no FUDR		
	CT D3	CT D9	Significance	CT D3	CT D7	Significance
Number of Right 5° branches	2.0 (*n* = 58)	20.0 (*n* = 58)	****	13.0 (*n* = 44)	28.0 (*n* = 29)	****
Number of Left 5° branches	2.0 (*n* = 47)	16.0 (*n* = 42)	****	10.50 (*n* =50)	27.00 (*n* = 41)	****
Number of Right 6° branches	1.0 (*n* = 58)	5.5 (*n* = 58)	****	1.0 (*n* = 44)	5.0 (*n* = 29)	****
Number of Left 6° branches	1.0 (*n* = 47)	5.5 (*n* = 42)	****	1.0 (*n* = 50)	5.0 (*n* = 41)	****
Number of Dorsal menorahs	16.5 (*n* = 10)	20.0 (*n* = 11)	ns	19.0 (*n* = 21)	16.0 (*n* = 19)	**
Number of Ventral menorahs	17.0 (*n* = 20)	16.0 (*n* = 14)	ns	16.5 (*n* = 22)	16.0 (*n* = 39)	ns
Number of Anterior menorahs	12.5 (*n* = 30)	13.0 (*n* = 25)	ns	13.0 (*n* = 43)	11.0 (*n* = 58)	***
Number of Posterior menorahs	5.0 (*n* = 30)	5.0 (*n* = 25)	ns	5.0 (*n* = 43)	5.0 (*n* = 58)	ns
Number of Dorsal 4° branches	66.0 (*n* = 10)	78.0 (*n* = 11)	ns	67.0 (*n* = 21)	68.0 (*n* = 19)	ns
Number of Ventral 4° branches	66.0 (*n* = 20)	72.5 (*n* = 14)	ns	77.0 (*n* = 22)	75.0 (*n* = 39)	ns
Number of Anterior 4° branches	40.0 (*n* = 30)	44.0 (*n* = 25)	*	47.0 (*n* = 43)	45.0 (*n* = 58)	ns
Number of Posterior 4° branches	26.0 (*n* = 30)	29.0 (*n* = 25)	ns	25.0 (*n* = 43)	25.0 (*n* = 58)	ns
Number of Dorsal 5° branches/ Dorsal menorah	0.5045 (*n* = 10)	2.000 (*n* = 11)	***	0.0625 (*n* = 21)	0.3158 (*n* = 19)	****
Number of Ventral 5° branches/ Ventral menorah	0.1563 (*n* = 20)	0.3643 (*n* = 14)	****	0.0816 (*n* = 22)	0.3571 (*n* = 39)	****
Number of Anterior 5° branches/ Anterior menorah	0.2020 (*n* = 30)	1.1670 (*n* = 25)	****	0.0000 (*n* = 43)	0.2000 (*n* = 58)	****
Number of Posterior 5° branches/ Posterior menorah	1.3750 (*n* = 30)	3.0000 (*n* = 25)	****	0.1667 (*n* = 43)	0.6333 (*n* = 58)	****
Number of Dorsal 5° branches/ Dorsal 4° branches	0.1257 (*n* = 10)	0.4103 (*n* = 11)	***	0.0161 (*n* = 21)	0.0641 (*n* = 19)	***
Number of Ventral 5° branches/ Ventral 4° branches	0.1563 (*n* = 20)	0.3643 (*n* = 14)	****	0.0160 (*n* = 22)	0.0806 (*n* = 39)	****
Number of Anterior 5° branches/ Anterior 4° branches	0.0519 (*n* = 30)	0.3056 (*n* = 25)	****	0.0000 (*n* = 43)	0.0454 (*n* = 58)	****
Number of Posterior 5° branches/ Posterior 4° branches	1.3750 (*n* = 30)	3.0000 (*n* = 25)	****	0.0357 (*n* = 43)	0.1200 (*n* = 58)	****
Number of Dorsal 6° branches/ Dorsal menorah	0.0000 (*n* = 10)	0.2500 (*n* = 11)	**	Refer to Figure 1		
Number of Ventral 6° branches/ Ventral menorah	0.0000 (*n* = 20)	0.1333 (*n* = 14)	***			
Number of Anterior 6° branches/ Anterior menorah	0.0000 (*n* = 30)	0.0909 (*n* = 25)	****			
Number of Posterior 6° branches/ Posterior menorah	0.0000 (*n* = 30)	0.4000 (*n* = 25)	****			
Number of Dorsal 6° branches/ Dorsal 4° branches	0.0000 (*n* = 10)	0.0635 (*n* = 11)	**			
Number of Ventral 6° branches/ Ventral 4° branches	0.0000 (*n* = 20)	0.0329 (*n* = 14)	**			
Number of Anterior 6° branches/ Anterior 4° branches	0.0000 (*n* = 30)	0.0238 (*n* = 25)	****			
Number of Posterior 6° branches/ Posterior 4° branches	0.0000 (*n* = 30)	0.0689 (*n* = 25)	****			

Table S2

Correlation	with FUDR			no FUDR		
	p value	*r* value	*n*	*p* value	*r* value	*n*
Dorsal 5° branches – Mean Wavelength	0.0613	-0.2666	49	***	-0.5883	34
Ventral 5° branches – Mean Wavelength	ns	-0.0218	30	ns	-0.1296	44
Dorsal 5° branches – Mean Amplitude	ns	-0.2369	49	***	-0.5878	34
Ventral 5° branches – Mean Amplitude	ns	0.0913	30	ns	-0.2042	44
Dorsal 5° branches – Wavelength CV	**	0.3706	49	****	0.6785	34
Ventral 5° branches – Wavelength CV	ns	0.2933	30	*	0.3375	44
Dorsal 5° branches – Amplitude CV	0.0829	0.2505	49	***	0.5494	34
Ventral 5° branches – Amplitude CV	ns	-0.0141	30	**	0.3947	44
Dorsal 6° branches – Mean Wavelength	0.0747	-0.2570	49	*	-0.3517	34
Ventral 5° branches – Mean Wavelength	ns	0.0631	30	ns	0.2477	44
Dorsal 6° branches – Mean Amplitude	**	-0.3656	49	0.0721	-0.3123	34
Ventral 6° branches – Mean Amplitude	ns	0.2818	30	0.0622	-0.2835	44
Dorsal 6° branches – Wavelength CV	**	0.5012	49	*	0.3714	34
Ventral 6° branches – Wavelength CV	ns	0.1033	30	***	0.5239	44
Dorsal 6° branches – Amplitude CV	****	0.5731	49	**	0.4680	34
Ventral 6° branches – Amplitude CV	ns	-0.0747	30	**	0.4649	44
5° branches – Mean Wavelength	ns	-0.1710	79	Refer to Figure 2		
5° branches – Mean Amplitude	ns	-0.1222	79			
5° branches – Wavelength CV	**	0.3380	79			
5° branches – Amplitude CV	ns	0.1540	79			
6° branches – Mean Wavelength	0.0623	-0.2107	79	*	-0.2888	78
6° branches – Mean Amplitude	ns	-0.1338	79	**	-0.3031	78
6° branches – Wavelength CV	**	0.3402	79	****	0.4720	78
6° branches – Amplitude CV	**	0.3343	79	****	0.4731	78

Table S3: Strain information

Strain	Genotype	Notes
N2	Wild type	CGC
BC10008	*dpy-5(e907) I; sEx10008*	CGC, used to create ELZ23
CB128	*dpy-10(e128) II;*	CGC, used to create ELZ43
CZ10175	*zdIs5 [mec-4p::GFP + lin-15(+)] I*	CGC
ELZ23	*dpy-5(e907) I; F49H12.4::GFP; unc-119(+)(wdIs51)X*	-
ELZ30	*zdIs5 [mec-4p::GFP + lin-15(+)] I; dpy-5(e907) I*	-
ELZ43	*dpy-10(e128) II; F49H12.4::GFP; unc-119(+)(wdIs51) X*	-
ELZ55	*F49H12.4::GFP; unc-119(+)(wdIs51) X*	1x outcross of NC1686
ELZ60	*col-120(sy1526)IV; F49H12.4::GFP; unc-119(+)(wdIs51)X*	-
ELZ86	*F49H12.4::GFP;unc-119(+)(wdIs51)X; col-19p::dpy-5 gDNA (lxyEx24)*	100 ng/μL PELZ19 into ELZ55 (*dpy-5* overexpression line)
ELZ90	*col-120(sy1526)IV; F49H12.4::GFP; unc-119(+)(wdIs51)X; dpy7p::col120+ col-19p::col120(lxyEx26)*	20ng/μL PELZ31 + 20 ng/μL PELZ37 + 50 ng/μL PELZ5 into ELZ60 (*col-120* epidermis rescue line 2)
ELZ121	*ser-2(3)p::EMTB::GFP + ser-2(3)p::mCherry-PH(lxyEx48)*	4ng/μL PNYL931 + 4ng/μL PNYL720 + 50ng/μL PELZ5 into N2
ELZ124	*ser-2(3)p::GFP::UtrCH + ser-2(3)p::mCherry-PH(lxyEx51)*	4 ng/μL PNYL930 + 6ng/μL PNYL720 + 50 ng/μL PELZ5 into N2
ELZ132	*dpy-5(e907)I; F49H12.4::GFP; unc-119(+)(wdIs51)X; dpy-7p::dpy5(lxyEx57)*	30 ng/uL PELZ75 + 50 ng/uL PELZ5 into ELZ23 (*dpy-5* epidermis rescue line 2)
ELZ133	*dpy-5 (e907)I; F49H12.4::GFP;unc-119(+)(wdIs51)X; dpy-7p::dpy-5 cDNA (lxyEx58)*	30 ng/μL PELZ75 + 50 ng/μL PELZ5 into ELZ23 (*dpy-5* epidermis rescue line 1)
ELZ139	*mec-4(u253)X; F49H12,4::GFP;unc-119(+)(wdIs51)X*	-
ELZ150	*col-120(sy1526)IV; F49H12.4::GFP; unc-119(+)(wdIs51)X; col-19p::col-120 (lxyEx73)*	10 ng/uL PELZ37+ 50 ng/uL PELZ5 into ELZ60 (*col-120* epidermis rescue line 2)
ELZ155	*rig-3(ok2156) X*	4X outcross of RB1712
ELZ161	*ser-2(3)p::GFP (lxyEx77)*	10 ng/μL PELZ47+ 50 ng/μL PELZ4 into N2
ELZ166	*rig-3(ok2156)X; ser-2(3)p::GFP (lxyEx77)*	ELZ161 crossed with ELZ155
ELZ170	*ser-2(3)p::GFP (lxyEx77); col-19p::dpy-5 (lxyEx24)*	ELZ161 crossed with ELZ86
ELZ171	*rig-3(ok2156)X; ser-2(3)p::GFP (lxyEx77); col-19p::dpy-5 (lxyEx24)*	ELZ166 crossed with ELZ86
ELZ174	*rig-3(ok2156)X; ser-2(3)p::GFP (lxyEx77); unc-33p::rig-3 (lxyEx85)*	10 ng/uL PELZ86 + 10 ng/uL PNYL918 into ELZ166 (*rig-3* pan-neuronal rescue line)
ELZ177	*dpy-5(e907) I*	3x outcross of BC10008
ELZ178	*col-120(sy1526)IV*	3x outcross of PS8819
ELZ181	*dpy-5(e907)I; F49H12;4::GFP; unc-119(+)(wdIs51)X; dpy-5p::dpy5 (lxyEx89)*	5 ng/uL PELZ81 + 50 ng/uL PELZ5 into ELZ23 (*dpy-5* endogenous rescue line)
ELZ188	*F49H12.4::GFP; unc-119(+)(wdIs51) X; col-19p::col-120 (lxyEx95)*	30 ng/uL PELZ91 + 50 ng/uL PELZ5 into ELZ55 (*col-120* overexpression line 2)
ELZ190	*F49H12.4::GFP; unc-119(+)(wdIs51) X; col-19p::col-120 (lxyEx97)*	30 ng/uL PELZ91 + 50 ng/uL PELZ5 into ELZ55 (*col-120* overexpression line 1)
ELZ198	*col-120(sy1526)IV; F49H12.4::GFP; unc-119(+)(wdIs51)X; col-120p::col-120 (lxyEx105)*	5 ng/uL PELZ91 + 50 ng/uL PELZ5 into ELZ60

		(*col-120* endogenous rescue line)
ELZ204	*F49H12.4::GFP; unc-119(+)(wdIs51) X; unc-33p::rig-3 (lxyEx106)*	50 ng/uL PELZ86 + 50 ng/uL PELZ5 into ELZ55<br>(*rig-3* overexpression line)
ELZ207	*rig-3(ok2156)X; ser-2(3)p::GFP (lxyEx77); nmr-1p::rig-3 (lxyEx108)*	10 ng/uL PELZ97 + 10 ng/uL PNYL918 into ELZ166<br>(*rig-3* interneuron rescue line)
ELZ223	*mev-1(kn1) III; F49H12.4::GFP; unc-119(+)(wdIs51) X*	TK22 crossed with ELZ55
ELZ225	*zdIs5 [mec-4p::GFP + lin-15(+)] I; col-120(sy1526) IV*	CZ10175 crossed with ELZ178
ELZ226	*ser-2(3)p::GFP (lxyEx77)*; *col-19p::col-120 (lxyEx97)*	ELZ190 crossed with ELZ166 and selected animals without *ok2156*
ELZ227	*rig-3(ok2156)X*; *ser-2(3)p::GFP (lxyEx77)*; *col-19p::col-120 (lxyEx97)*	ELZ190 crossed with ELZ166
ELZ228	*daf-16(mu86)I; F49H12,4::GFP; unc-119(+)(wdIs51)X*; *col-19p::col-120 (lxyEx97)*	ELZ190 crossed with NYL2016
ELZ232	*daf-16(mu86)I; F49H12,4::GFP; unc-119(+)(wdIs51)X; col-19p::dpy-5 (lxyEx24)*	ELZ86 crossed with NYL2016
ELZ233	*dpy-5(e907)I; F49H12.4::GFP; unc-119(+)(wdIs51) X; unc-33p::rig-3 (lxyEx106)*	ELZ204 crossed with ELZ177
ELZ240	*ser-2(3)p::GFP (lxyEx77)*	1X outcross of ELZ161
ELZ242	*col-120(sy1526) IV; F49H12.4::GFP;unc-119(+)(wdIs51)X; unc-33p::rig-3(lxyEx106)*	ELZ204 crossed with ELZ178
ELZ243	*col-141(gk5185)V; F49H12.4::GFP;unc-119(+)(wdIs51)X*	VC4094 crossed with ELZ55
NC1686	*F49H12.4::GFP; unc-119(+)(wdIs51)X*	CGC, used to create ELZ55
NYL1620	*daf-2(e1370)III; F49H12,4::GFP; unc-119(+)(wdIs51)X*	
NYL2016	*daf-16(mu86)I; F49H12,4::GFP; unc-119(+)(wdIs51)X*	
PS8819	*col-120(sy1526)IV*	CGC, used to create ELZ60
RB1712	*rig-3(ok2156) X*	CGC, used to create ELZ155
TJ356	*zIs356 [daf-16p::DAF-16a/b::GFP; rol-6] IV*	CGC
TK22	*mev-1(kn1) III*	CGC, used to create ELZ223
VC4094	*col-141(gk5185) V*	CGC, used to create ELZ243

Table S4

Plasmid number	Plasmid description
PELZ4	*ttx-3p::GFP*
PELZ5	*ttx-3p::RFP*
PELZ19	*col-19p::dpy-5 cDNA*
PELZ23	*L4440::dpy-5 cDNA*
PELZ31	*dpy-7p::col-120*
PELZ32	*dpy-30p::col120 cDNA*
PELZ37	*col-19p::col-120 cDNA*
PELZ44	*L4440::col-120*
PELZ45	*L4440::col-120* (*col-120* RNAi clone 1)
PELZ46	*L4440::col-120* (*col-120* RNAi clone 2)
PELZ47	*ser-2(3)p::GFP*
PELZ48	*L4440::dpy-5* (*dpy-5* RNAi clone 2)
PELZ50	*L4440::pat-2*
PELZ75	*dpy-7p (unc-54 3'UTR)::dpy-5*
PELZ81	*dpy-5p::dpy-5*
PELZ86	*unc-33p::rig-3*
PELZ88	*L4440::dpy-5* (*dpy-5* RNAi clone 1)
PELZ91	*col-120p::col-120*
PELZ97	*nmr-1p::rig-3*
PNYL720	*ser-2(3)p::mCherry-PH*
PNYL918	*arl-13p::GFP*
PNYL930	*ser-2(3)p::GFP-UtrCH*
PNYL931	*ser-2(3)p::EMTB::GFP*
C. elegans RNAi feeding library, Horizon Discovery	*L4440::rig-3*
C. elegans RNAi feeding library, Horizon Discovery	*L4440::ina-1*
C. elegans RNAi feeding library, Horizon Discovery	*L4440::ddr-2*
C. elegans RNAi feeding library, Horizon Discovery	*L4440::sdn-1*
C. elegans RNAi feeding library, Horizon Discovery	*L4440::rol-6*
C. elegans RNAi feeding library, Horizon Discovery	*L4440::col-129*

Table S5

Primer name	Primer sequence (5'-3')	Purpose
ok2156 Forward 1.4	CGTCATTTCATCAGAAGCCATG	Forward genotyping primer for *rig-3(ok2156)*
ok2156 Reverse 1.2	CCTGTAATAGAGTCGTTTACAACTTGTTTC	Reverse genotyping primer for *rig-3(ok2156)*
ok2156 Reverse 2	GTCATCTGAATCATTGGTTCTTTAC	Reverse genotyping primer for *rig-3(ok2156)*
dpy-5-For	TTA GAC GCG TCT GCG CTT TCT CTC TG	Forward primer for *dpy-5* cDNA amplification
dpy-5-Rev	ATGGTAAAGGCCGTCGTCGGATTCG	Reverse primer for *dpy-5* cDNA amplification
dpy-5(e907)-F1	GAAGCATGGGTCTCGTTAG	Forward genotyping primer for *dpy-5*
dpy-5(e907)-F2	GTCCTGGAGTTCCTTGATC	Forward genotyping primer for *dpy-5*
dpy-5(e907)-R	GAAGGTGTGTCCTCATGG	Reverse genotyping primer for *dpy-5*
col-120 For	ATGAATTTGAAGAAGAATTTAGTGAATTCT GAAGAGGATG	Forward primer for *col-120* cDNA amplification
col-120 Rev	TTAGTATCCAGGAGCAGTTCGTGGAGGTG	Reverse primer for *col-120* cDNA amplification
col-120(sy2156)-F	GTTGCTTTCGTGGCGACTG	Forward genotyping primer for *col-120*
col-120(sy2156)-R	CCTCCATATCCAGCTTGTC	Reverse genotyping primer for *col-120*
rig-3 For	ATGGGACGACTACTTGCCAAGATG	Forward primer for *rig-3* cDNA amplification
rig-3 Rev	TTAGATAAAAAGACAGACAAAAAATAACGT G	Reverse primer for *rig-3* cDNA amplification
pat-2 For	CCTTTTGGGAACTCAAGACAATACAATGCT TATCAATG	Forward primer for *pat-2* cDNA amplification
pat-2 Rev	TTATAGCATTTGTCCGTGACGTCCCTGATT GTACTG	Reverse primer for *pat-2* cDNA amplification
ama-1 Forward	ATCTGTGCAAAGCCAAGTCG	qPCR Forward primer for *ama-1*
ama-1 Reverse	CCATTCTGCGTTGATGTCGA	qPCR Reverse primer for *ama-1*
cdc-42 Forward	GTTTGCTTCTCCGTGGTTG	qPCR Forward primer for *cdc-42*
cdc-42 Reverse	GCCAGTTTCTCGAGCATTC	qPCR Reverse primer for *cdc-42*
col-120 Forward	GGAGCTTCTTCCCCCTCTCG	qPCR Forward primer for *col-120*
col-120 Reverse	GGCCCAGCAAGACCAATTCC	qPCR Reverse primer for *col-120*
dpy-3 Forward	CAATCACGTACAGCATCTTC	qPCR Forward primer for *dpy-3*
dpy-3 Reverse	GTAGCAGACTGGACGGTGAC	qPCR Reverse primer for *dpy-3*
dpy-5 Forward	GTTCGTGGAATCTTCGGACG	qPCR Forward primer for *dpy-5*
dpy-5 Reverse	CCGGCTGGGCATCCTTGAG	qPCR Reverse primer for *dpy-5*
col-10 Forward	CTTGTAACCCTCTCTACC	qPCR Forward primer for *col-10*
col-10 Reverse	CATCGAGAACCTCATCGTG	qPCR Reverse primer for *col-10*
